# Supplementary material for: Optical tissue clearing and machine learning can precisely characterize extravasation and blood vessel architecture in brain tumors
Source: Commun Biol. 2021 Jul 1;4:815. doi: 10.1038/s42003-021-02275-y (PMC8249617; doi:10.1038/s42003-021-02275-y)
Supplement: Supplementary file 2 — Supplementary Information [file 42003_2021_2275_MOESM2_ESM.pdf]

Supplementary Information

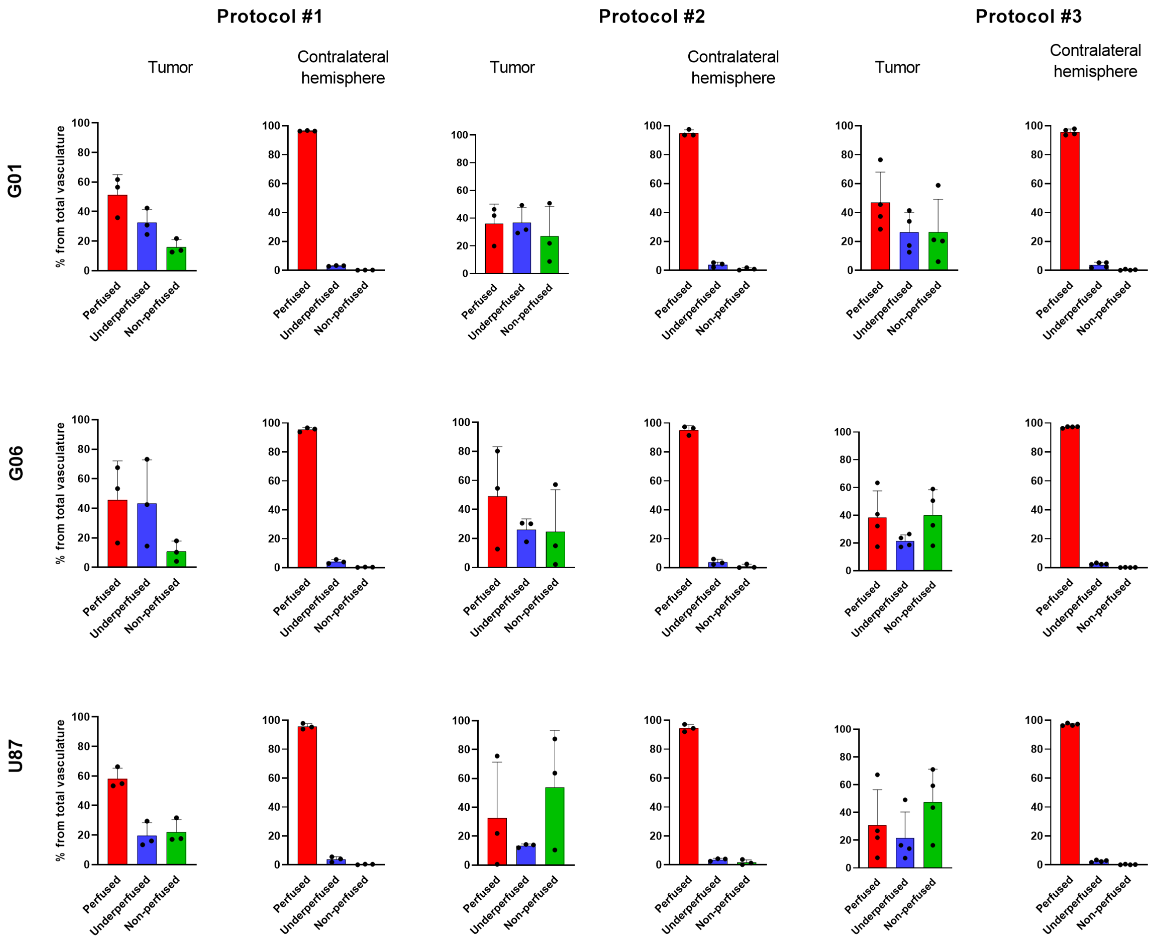

Supplementary Figure 1. Quantification of transcardial perfusion degree in different GBM models under different experimental conditions. The proportion of perfused vessels was comparable in all tumor models (P > 0.6) and changing the experimental protocol did not have any effect on proportion of perfused vessels (P > 0.9). Similar result was noted for underperfused vessels (P > 0.7 for different tumor models and P > 0.6 for different perfusion protocols), as well as for the proportion of non-perfused vessels (P > 0.3 for different tumor models and P > 0.4 for different perfusion protocols). The contralateral tumor-free hemisphere showed high degree of transcardial perfusion regardless of the tumor model or perfusion protocol (P > 0.9). n = 3 mice per tumor model for groups perfused according to the protocols 1 and 2, n = 4 mice per tumor model for groups perfused according to the protocol 3. Significance is calculated by logistic regression after arcsin conversion of percentages. Data are presented as mean ± SD. Dots represent the values for a given vessel class in one mouse.

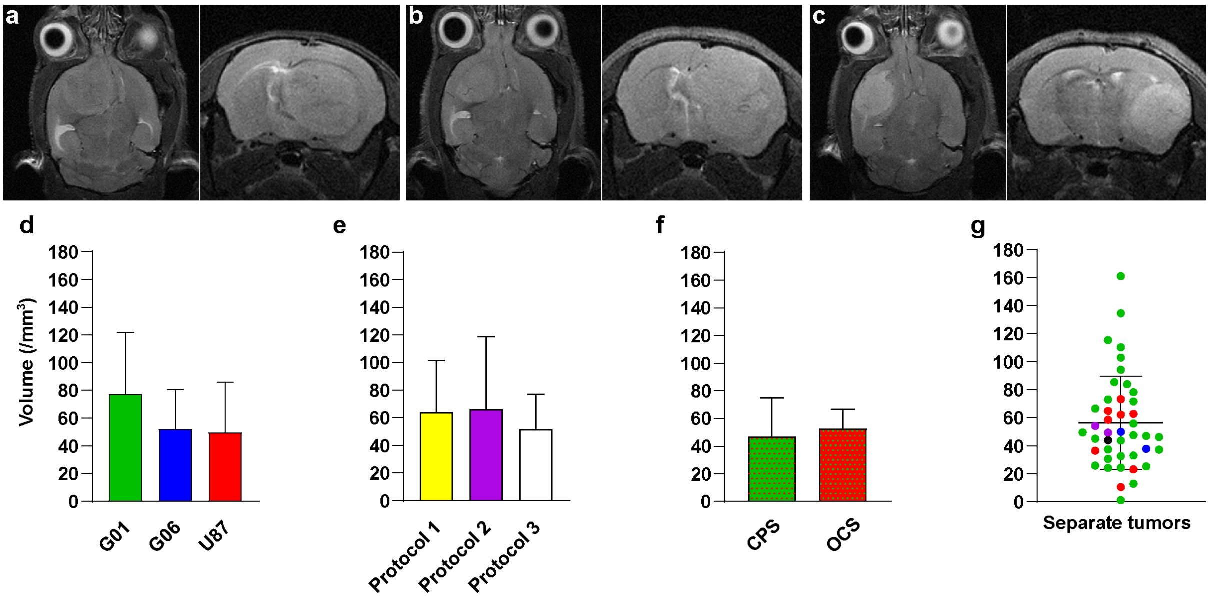

Supplementary Figure 2. MRI data and volumes of the tumor used in the study. Representative horizontal and coronal MRI sections of mouse brains with tumors arising from G01 (**a**), G06 (**b**) and U87 (**c**) cell lines taken within 24 hours before the endpoint. (**d**) Tumor volume for the groups bearing tumors arising from different cell lines, (n = 10 per group, P = 0.2, One-way ANOVA). Data are presented as mean ± SD. (**e**) Tumor volume for the groups undergoing perfusion assessment with different protocols (n = 9 for protocol 1 and 2, n = 12 for protocol 3, P = 0.65, One-way ANOVA). Data are presented as mean ± SD. (**f**) Volume of the tumors used for comparing microscopy method performance, (n = 5 for conventionally prepared samples (CPS), n = 3 for optically cleared samples (OCS), P > 0.9999, Mann-Whitney test). Data are presented as mean ± SD. (**g**) Volumes of separate GBM tumors used in study (color coding: green depicts tumors used for assessment of transcardial perfusion degree, blue depicts tumors used for control experiments in transcardial perfusion assessment without TRITC-dextran injection, red depicts tumors used for comparing the performance of conventional tissue preparation and optical clearing for extravasation analysis, magenta depicts tumors used for imaging of transcardial perfusion deficiency signs with transmission electron microscopy, black depicts tumor used for analysis of TRITC-albumin extravasation and angioarchitecture). Error bar is mean ± SD.

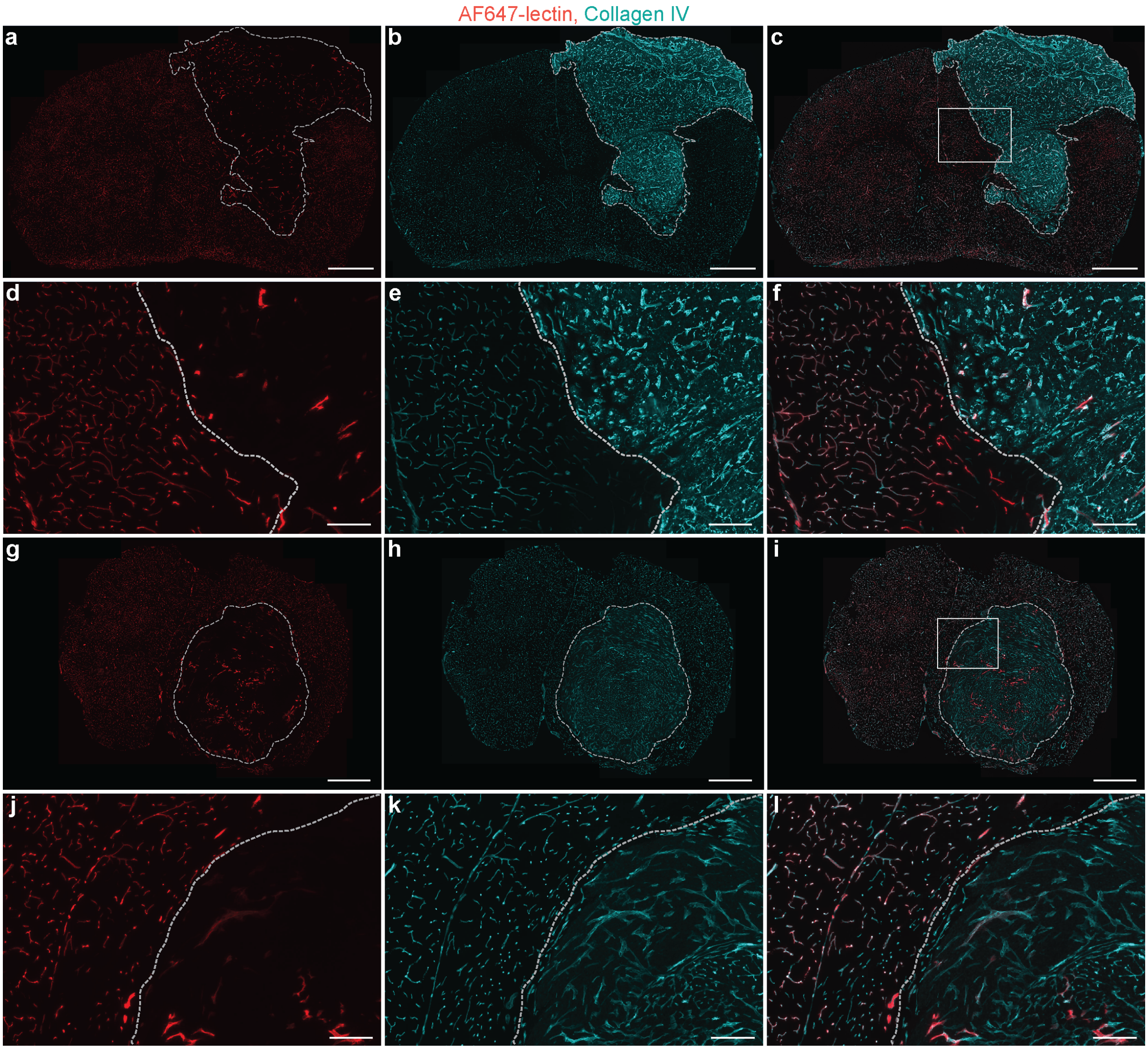

Supplementary Figure 3. Restriction of the perfusate flow in the GBM vasculature is not caused by prior TRITC-dextran injection. Representative images of brain coronal sections from a mouse bearing a tumor arising from the G01 cell line depicting vessels labeled by IV injection of AF647-lectin (**a**) and collagen IV immunostaining (**b**). (**c**) Overlay image showing deficiency of AF647-lectin penetration to the tumor vasculature in the G01 model. Scale bars, 1000 µm. (**d-f**) High magnification images of the region marked in (c). Scale bars, 200 µm. Representative images of brain coronal sections from a mouse bearing a tumor arising from the U87 cell line depicting vessels labeled by IV injection of AF647-lectin (**g**) and collagen IV immunostaining (**h**). (**i**) Overlay image showing deficiency of lectin penetration to the tumor vasculature in U87 model. Scale bars, 1000 µm. (**j-l**) High magnification images of the region marked in (i). Scale bars, 200 µm. Tumor borders are highlighted with dashed lines.

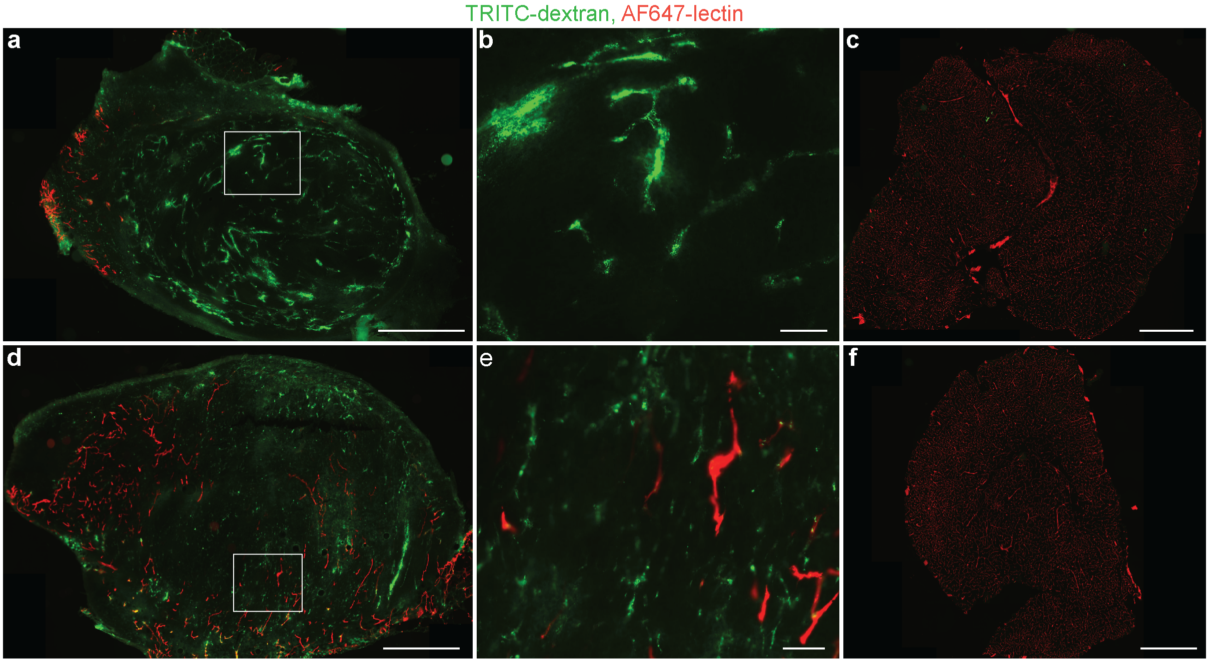

Supplementary Figure 4. Incomplete transcardial perfusion in two syngeneic colorectal cancer models. Representative slide-scanned images of tumors arising from the MC38 (**a**) and CT26 (**d**) cell lines dissected from the animals after dextran injection and perfusion with lectin. Scale bars, 1000 µm. (**b, e**) High magnification images of the regions marked in (a, d). Scale bars, 100 µm. (**c, f**) Slide-scanned images of the brain taken from the same animals depicting proper perfusion degree in the brain vessels. Scale bars, 1000 µm.

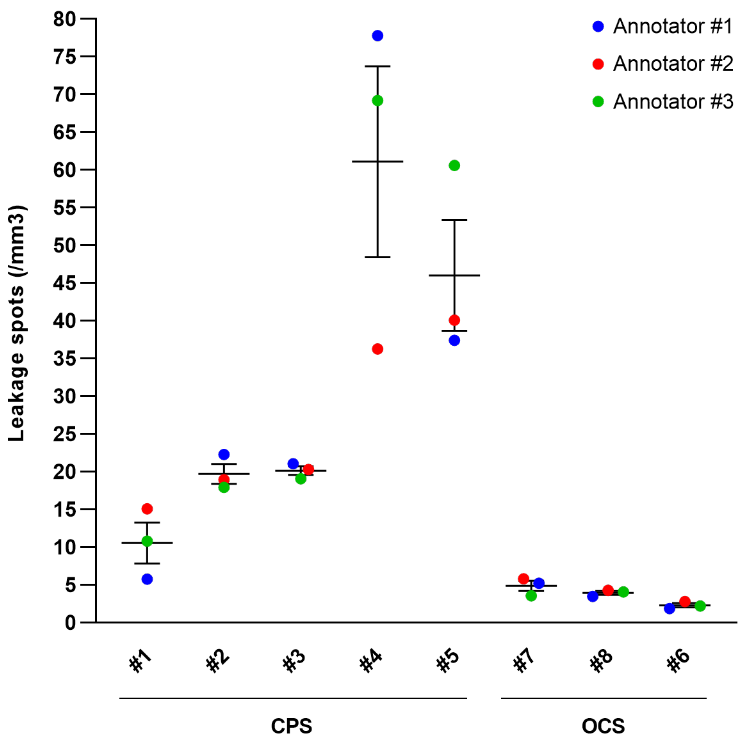

Supplementary Figure 5. Quantification of extravasation spots done by three annotators in conventionally prepared and optically cleared samples. Data are presented as separate results obtained by each researcher for each tumor, error bars represent mean ± SEM. CPS, conventionally prepared samples, OCS, optically cleared samples.

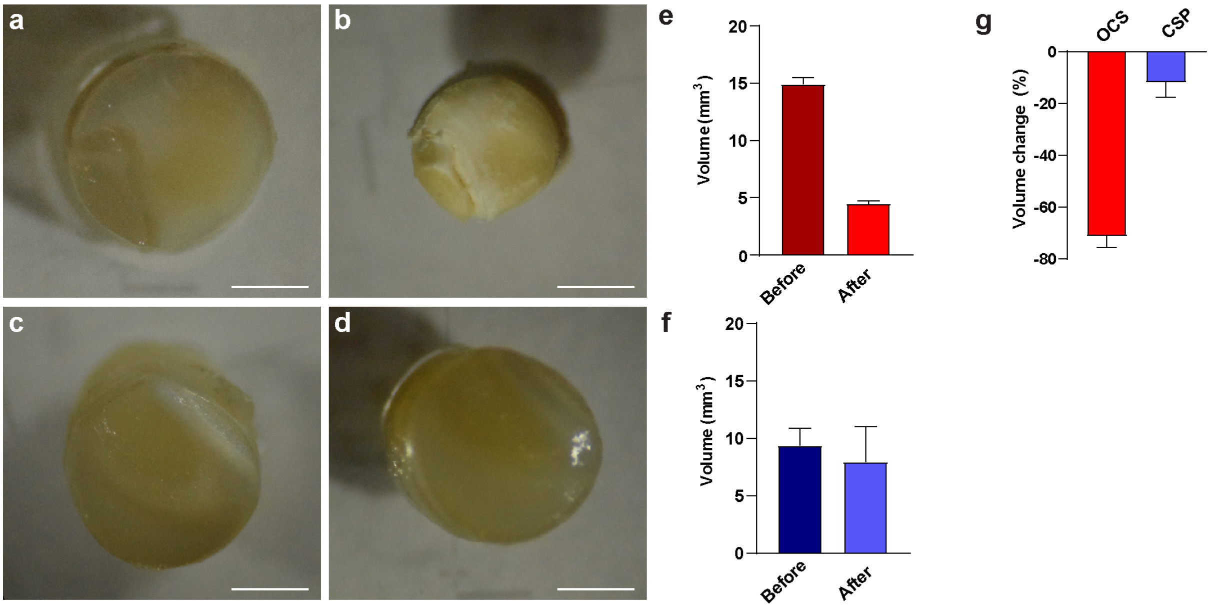

Supplementary Figure 6. Tissue volume shrinkage after dehydration or cryopreservation. (**a, c**) Stereomicroscopic images of samples taken from a brain after perfusion-fixation and overnight postfixation in 4 % formaldehyde. (**b**) The sample from (a) after dehydration. (**d**) The sample from (c) after cryopreservation with sucrose. Scale bars, 1000 µm. (**e**) Sample volume before and after dehydration (n = 3). Data are presented as median ± interquartile range. (**f**) Sample volume before and after undergoing cryopreservation (n = 3). Data are presented as median ± interquartile range. (**g**) Percentage of volume change for samples after dehydration and cryopreservation. Data are presented as median ± interquartile range.

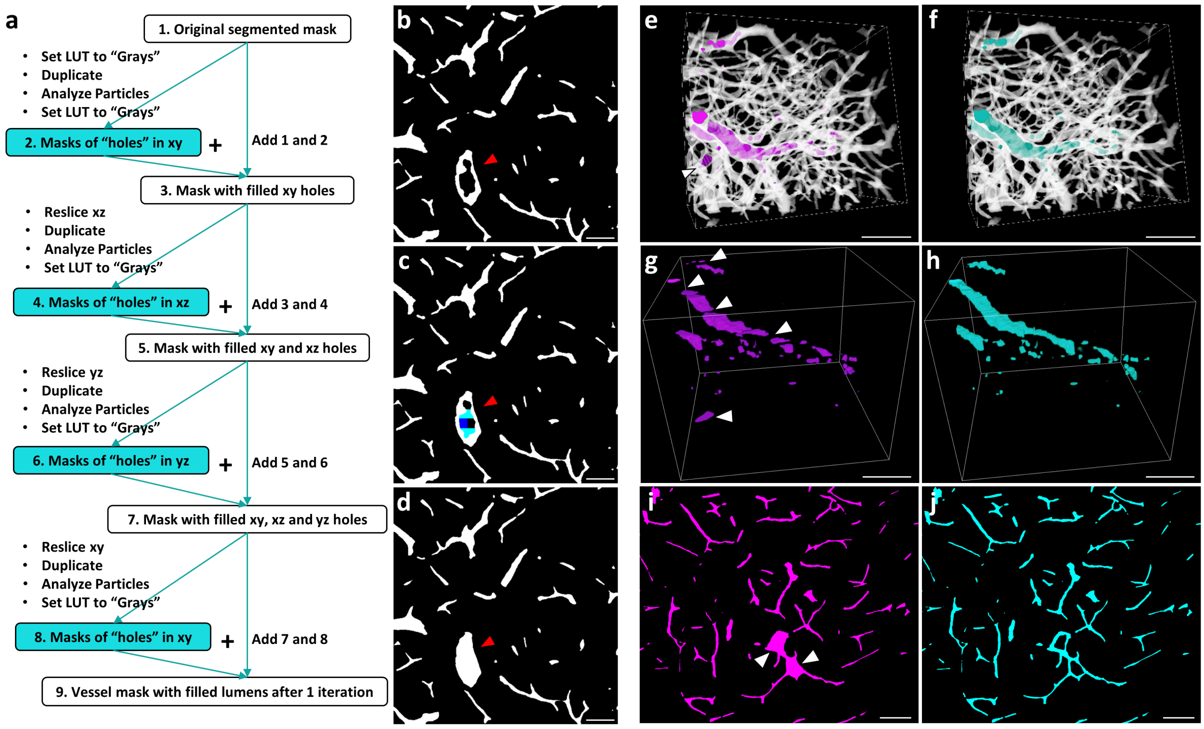

Supplementary Figure 7. Performance of the in-house developed workflow for the adjustable vessel lumen filling. (**a**) Schematic depiction of one iteration of the developed workflow for the adjustable filling of the vessel lumens. (**b**) Binary mask of the vessels after filling holes operation in the xy plane demonstrating the lumen above established size limit not being filled. (**c**) Binary mask of the vessels after filling holes in xz plane (cyan) and yz plane (blue). (**d**) Binary mask of the vessels after the first iteration of the lumen filling workflow and 2D median filtering demonstrating the lumen above established size limit being filled. Scale bars, 50 µm. In (b-d) an arrow points at the large vessel segmented as a hollow tube. 3D rendering of the vasculature (white) with lumen filled using standard "fill hole" operation in Fiji (magenta) (**e**), and using developed workflow (cyan) (**f**), an arrow points on the space between small vessels being erroneously filled. Binary mask of the vessel lumens produced by standard "fill hole" operation in Fiji (**g**), and developed workflow (**h**), arrows point on the imprecisions in the mask produced by standard "fill hole" operation. Binary masks of the vessels after lumen filling done using standard "fill hole" operation in Fiji (**i**) and developed workflow (**j**) arrows point on the spaces surrounded by small capillaries being filled by standard "fill hole" operation in Fiji. Scale bars, 100 µm.

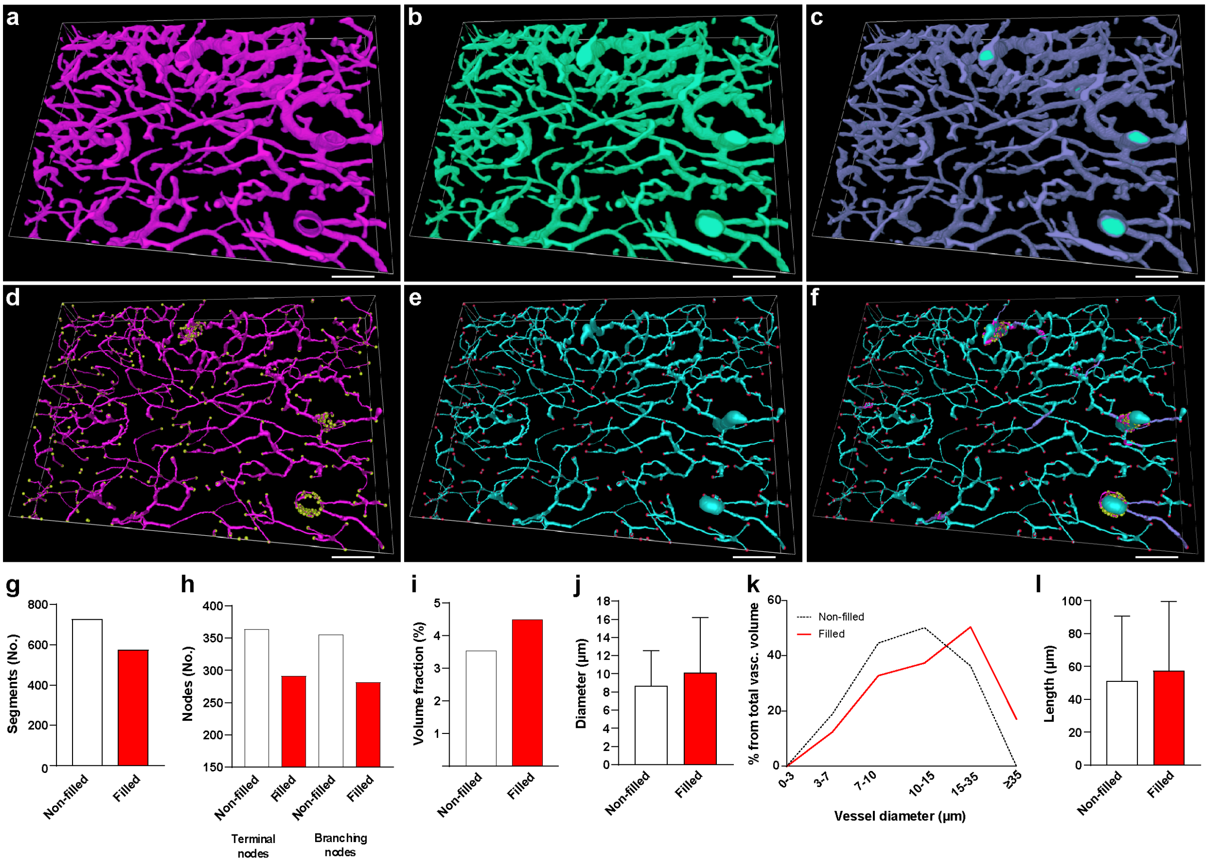

Supplementary Figure 8. Effect of vessel lumen filling in the postprocessing stage on the precision of large vessel recognition. (**a**) 3D rendering of segmented tumor vessels after undergoing all postprocessing steps except the lumen filling step. (**b**) 3D rendering of the dataset in (a) after undergoing all postprocessing steps including the lumen filling step. (**c**) Overlay of images (a) and (b). (**d**) Traced vasculature from dataset depicted in image (a) (magenta depicts segments, yellow depicts nodes). (**e**) Traced vasculature from dataset depicted in image (b) (cyan depicts segments, red depicts nodes). (**f**) Overlay of images (d) and (e). Scale bars, 100 µm. (**g**) Number of vascular segments. (**h**) Number of branching and terminal nodes. (**i**) Volume fraction of the vasculature. (**j**) Mean vessel diameter. Data are presented as mean ± SD. (**k**) Histogram representing the volume fraction occupied by vessels of a certain diameter from the total vascular volume fraction. (**l**) Mean length of the vascular segment. Data are presented as mean ± SD.

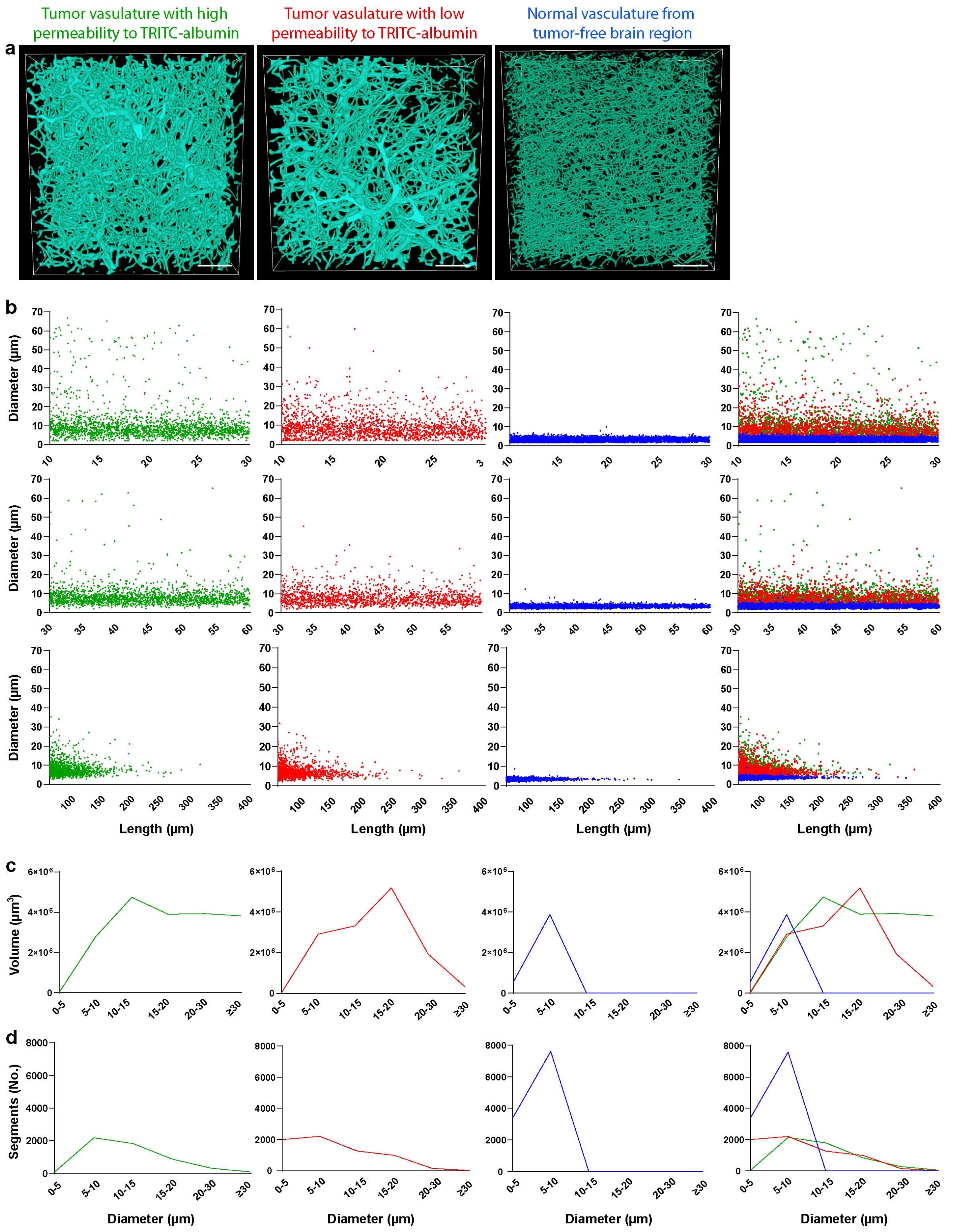

Supplementary Figure 9. High-resolution multidimensional analysis of angioarchitecture. (a) 3D rendering of representative image subsets from different tumor regions and tumor-free brain region after vasculature segmentation and postprocessing step. Scale bars, 100 µm. (**b**) Scatter plots relating the diameter and length of vessel segment in different tumor regions and tumor-free brain region. Histograms representing total volume (**c**) and total number of vessel segments (**d**) of vessels with certain diameter in different tumor regions and tumor-free brain region.

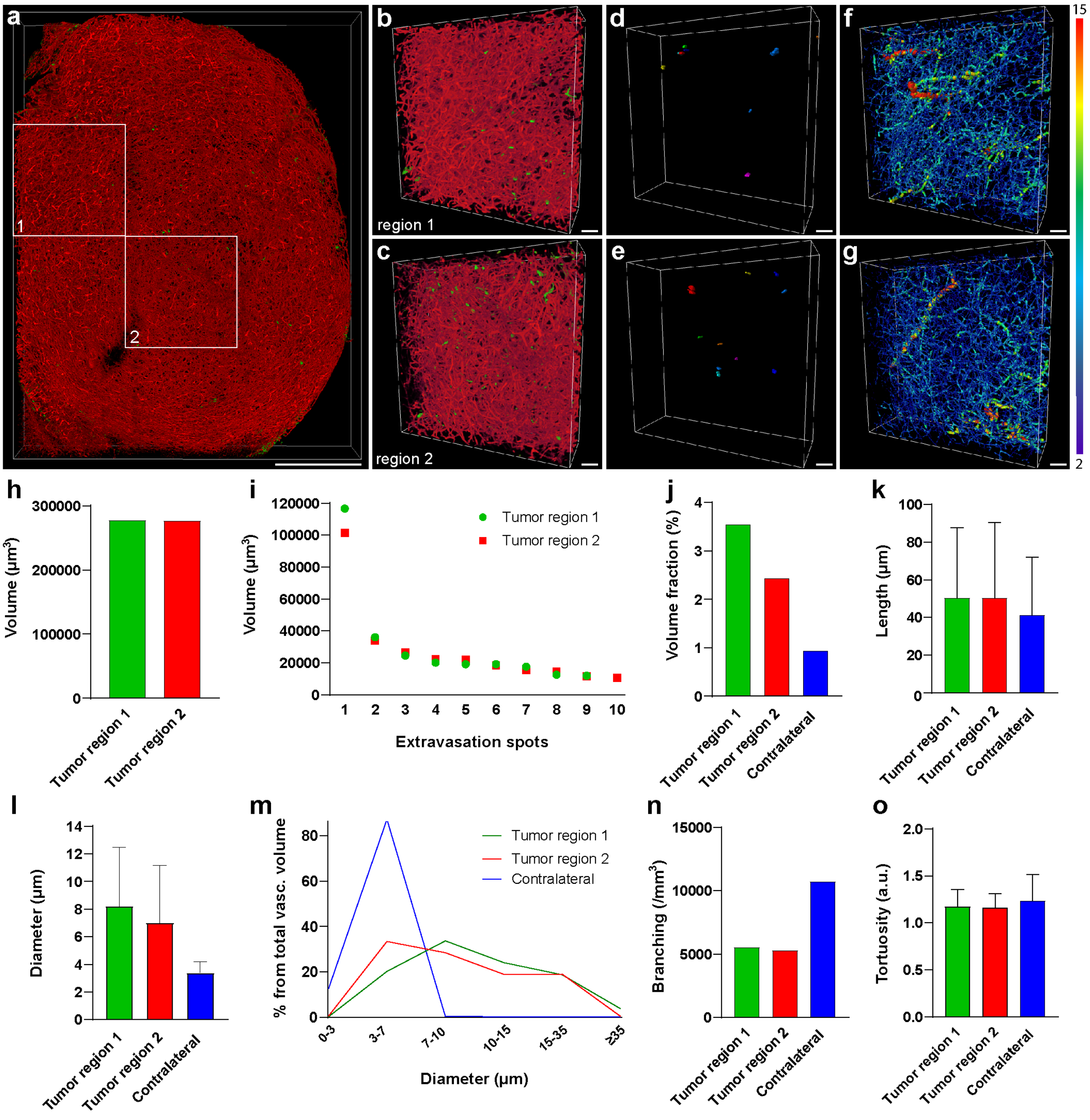

Supplementary Figure 10. Method application example: relationship between GBM vasculature permeability to TRITC-dextran and the angioarchitecture. (**a**) 3D rendering of a large data subset derived from a cleared tumor dissected from an animal after IV injection of TRITC-dextran (green) and AF647-lectin (red). Scale bar, 1000 µm. (**b-c**) 3D rendering of the regions marked in (a) in higher magnification. (**d-e**) Recognized extravasation spots from the subsets in (b-c) colored independently of any attributes (spots smaller than 10 000 µm^3 excluded from the mask). (**f-g**) traced vasculature from the subsets in (b-c) colored according to the vessel diameter, numbers for minimum and maximum vessel diameter are presented in µm. Scale bars, 100 µm. (**h**) Total volume of extravasation in the tissue subsets. (**i**) Volumes of the separate extravasation spots recognized in (b-c) after correction. (**j**) Vascular volume fraction in the tissue subsets and the tumor-free, contralateral hemisphere. (**k**) Mean length of the vascular segments in tissue subsets and the tumor-free, contralateral hemisphere. Data are presented as mean ± SD. (**l**) Mean diameter of the vascular segments in tissue subsets and the tumor-free, contralateral hemisphere. Data are

presented as mean ± SD. (**m**) Histogram representing the volume fraction occupied by vessels of a certain diameter from the total vascular volume fraction in the tissue subsets and the tumor-free, contralateral hemisphere. (**n**) Number of branching points per volume of tissue in the tissue subsets and the tumor-free, contralateral hemisphere. (**o**) Tortuosity of the vasculature in the tissue subsets and the tumor-free, contralateral hemisphere. Data are presented as mean ± SD.

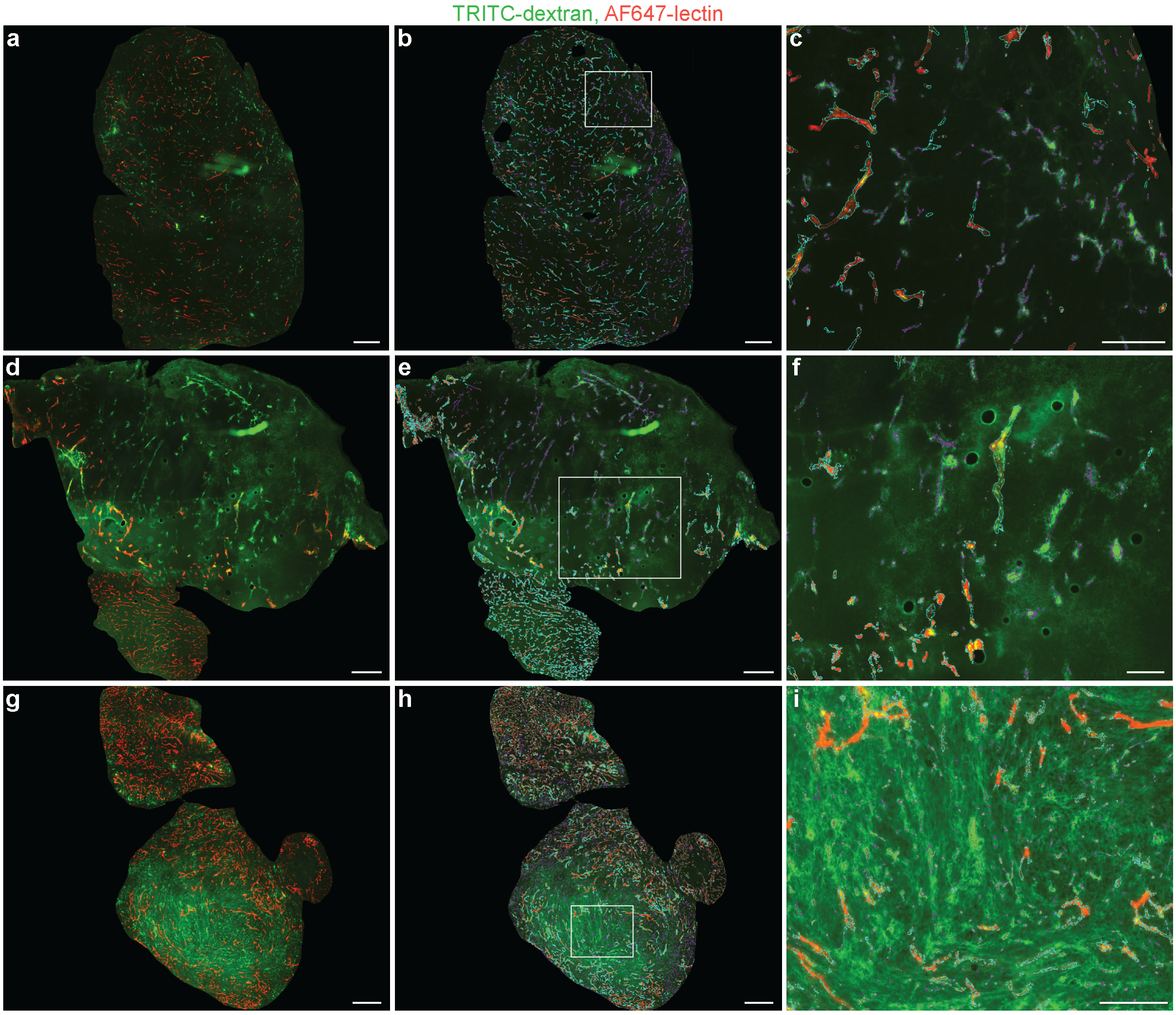

Supplementary Figure 11. Modification of original training model and extravasation spot deletion for images with higher than average TRITC background signal. Tumor areas selected from the slide-scanned coronal sections of brains from animals bearing tumors arising from the G01 (**a, d**) and U87 (**g**) cell lines with higher than average levels of TRITC-dextran extravasation causing a high TRITC background signal. (**b, e, h**) Regions depicted in (a, d, g) overlaid with segmentation masks produced by the modified models, (b) also depicts deletion of an especially high-intensity leakage spots which would otherwise be falsely segmented. Scale bars, 500 µm. (**c, f, i**) High magnification images of the areas marked in (b, e, h) illustrating correct segmentation results provided by the modified models despite the presence of high dextran extravasation levels. Segmentation color-coding: magenta depicts non-perfused vessels, cyan depicts underperfused vessels, orange depicts perfused vessels. Scale bars, 200 µm.
