# Supplementary material for: Optical tissue clearing and machine learning can precisely characterize extravasation and blood vessel architecture in brain tumors
Source: Commun Biol. 2021 Jul 1;4:815. doi: 10.1038/s42003-021-02275-y (PMC8249617; doi:10.1038/s42003-021-02275-y)
Supplement: Supplementary file 3 — Description of Additional Supplementary Files [file 42003_2021_2275_MOESM3_ESM.pdf]

Description of Additional Supplementary Files

File Name: Supplementary Data 1
Description: The excel file contains all the data underlying graphs and charts presented in the main figures. Each separate sheet corresponds to the figure panel it is named after.

File Name: Supplementary Movie 1
Description: **Visualizing extravasation analysis workflow.** The movie illustrates detection of extravasation spots in GBM by showing 3D reconstruction of deconvolved original dataset, and its overlays with the dataset after segmentation and after postprocessing stage. Dimensions of the bounding box: x = 691 µm, y = 424 µm, z = 240 µm.

File Name: Supplementary Movie 2
Description: **Visualizing vasculature analysis workflow.** The movie illustrates image processing stages of the vasculature analysis workflow and demonstrates the results of each operation as well as preservation of vasculature network integrity throughout the processing stages. Dimensions of the bounding box: x = 1078 µm, y = 1046 µm, z = 348 µm.
