# Supplementary material for: Optical tissue clearing and machine learning can precisely characterize extravasation and blood vessel architecture in brain tumors
Source: Commun Biol. 2021 Jul 1;4:815. doi: 10.1038/s42003-021-02275-y (PMC8249617; doi:10.1038/s42003-021-02275-y)
Supplement: Supplementary file 7 — Reporting Summary [file 42003_2021_2275_MOESM7_ESM.pdf]

nature research

Corresponding author(s): Casper Hempel, Thomas Lars Andresen

Last updated by author(s): May 10, 2021

Reporting Summary

Nature Research wishes to improve the reproducibility of the work that we publish. This form provides structure for consistency and transparency in reporting. For further information on Nature Research policies, see our Editorial Policies and the Editorial Policy Checklist.

Statistics

For all statistical analyses, confirm that the following items are present in the figure legend, table legend, main text, or Methods section.

n/a	Confirmed	
☐	☒	The exact sample size (n) for each experimental group/condition, given as a discrete number and unit of measurement
☐	☒	A statement on whether measurements were taken from distinct samples or whether the same sample was measured repeatedly
☐	☒	The statistical test(s) used AND whether they are one- or two-sided<br>*Only common tests should be described solely by name; describe more complex techniques in the Methods section.*
☐	☒	A description of all covariates tested
☐	☒	A description of any assumptions or corrections, such as tests of normality and adjustment for multiple comparisons
☐	☒	A full description of the statistical parameters including central tendency (e.g. means) or other basic estimates (e.g. regression coefficient) AND variation (e.g. standard deviation) or associated estimates of uncertainty (e.g. confidence intervals)
☐	☒	For null hypothesis testing, the test statistic (e.g. F, t, r) with confidence intervals, effect sizes, degrees of freedom and P value noted<br>*Give P values as exact values whenever suitable.*
☒	☐	For Bayesian analysis, information on the choice of priors and Markov chain Monte Carlo settings
☒	☐	For hierarchical and complex designs, identification of the appropriate level for tests and full reporting of outcomes
☒	☐	Estimates of effect sizes (e.g. Cohen's d, Pearson's r), indicating how they were calculated

Our web collection on statistics for biologists contains articles on many of the points above.

Software and code

Policy information about availability of computer code

Data collection	For collecting microscopy images with slide scanner, Zen Blue 2.3 (Zeiss Microscopy) was used. For collecting confocal microscopy images, Zen Black 2012 SP 5 (Zeiss Microscopy) was used. For the acquisition of the MR images, Paravision 360 (Bruker) was used.
Data analysis	MRI data was analyzed with Horos software (Horos Project). For operations like deconvolution, spatial graph generation, distance map generation and 3D object counting, Amira 6.5 (Thermo Fisher Scientific) was used. For stitching of images, except for those obtained from optically cleared samples, Zen Blue 3.0 (Zeiss Microscopy) was used. For machine learning-based segmentation, commercially available software Intellesis (operating within Zen Blue by Zeiss Microscopy) was used. Basic image processing operations like filtering and thresholding as well as stitching of imaging datasets from optically cleared samples was done using FIJI software. FIJI software was also used for developing the method for vessel lumen filling in 3D space. The method is described in the manuscript and the code is available upon a request. Statistical analysis was performed using GraphPad Prism 8.1.1 (GraphPad software) and R for Windows 2.12.1.

For manuscripts utilizing custom algorithms or software that are central to the research but not yet described in published literature, software must be made available to editors and reviewers. We strongly encourage code deposition in a community repository (e.g. GitHub). See the Nature Research guidelines for submitting code & software for further information.

Data

Policy information about availability of data

All manuscripts must include a data availability statement. This statement should provide the following information, where applicable:

- Accession codes, unique identifiers, or web links for publicly available datasets
- A list of figures that have associated raw data
- A description of any restrictions on data availability

All quantitative data which were the basis for the current study are included in either main text or supplementary information. Large size of the imaging data, which

comprise numerous large imaging datasets (both large 2D slide-scanner datasets as well as large 3D datasets from cleared tissue samples) entail difficulties in uploading to the repositories. Furthermore due to complexity image the analysis workflows used, imaging data also comprises numerous preprocessed images as well as binary masks. These data are available from corresponding authors on reasonable request.

Field-specific reporting

Please select the one below that is the best fit for your research. If you are not sure, read the appropriate sections before making your selection.

- [x] Life sciences
- [ ] Behavioural & social sciences
- [ ] Ecological, evolutionary & environmental sciences

For a reference copy of the document with all sections, see nature.com/documents/nr-reporting-summary-flat.pdf

Life sciences study design

All studies must disclose on these points even when the disclosure is negative.

Sample size	Sample sizes were chosen based on pilot experiments. Sample sizes are specified in figure legends.
Data exclusions	No data points were excluded.
Replication	Each reported experiment was conducted in multiple animals. Perfusion experments were also conducted in multiple glioblastoma models.
Randomization	The animals were assigned to the different groups through usage of random number generator.
Blinding	Blinding could not be achieved in parts of the study requiring manual annotations due to apparent differences (e.g. signal-to-background ratio) between datasets from optically cleared and conventionally prepared samples, thereby, the annotations were performed independently by three annotators, the data from each annotator is presented in the supplementary information.

Reporting for specific materials, systems and methods

We require information from authors about some types of materials, experimental systems and methods used in many studies. Here, indicate whether each material, system or method listed is relevant to your study. If you are not sure if a list item applies to your research, read the appropriate section before selecting a response.

Materials & experimental systems

n/a	Involved in the study	
[ ]	[x]	Antibodies
[ ]	[x]	Eukaryotic cell lines
[x]	[ ]	Palaeontology and archaeology
[ ]	[x]	Animals and other organisms
[x]	[ ]	Human research participants
[x]	[ ]	Clinical data
[x]	[ ]	Dual use research of concern

Methods

n/a	Involved in the study	
[x]	[ ]	ChIP-seq
[x]	[ ]	Flow cytometry
[x]	[ ]	MRI-based neuroimaging

Antibodies

Antibodies used	Goat anti-type IV collagen polyclonal antibody, supplier: SouthernBiotech, catalog # 1340-01, Lot # LH2915-MG07
Validation	The antibody was validated by the manufacturer SouthernBiotech with the validation information for frozen sections of mouse tissues present on their website (https://www.southernbiotech.com/PolyclonalDetails.aspx?catno=1340-01#&panel1-1&panel2-1) including references to multiple papers employing the antibody in the same way as was done for the present study. As an additional point of validation in the present study, co-localization of the antibody labelling in normal brain vessels and labelling of this vessels with WGA lectins introduced by transcardial perfusion (and thereby providing specific labelling for the non-leaky vasculature) can be seen.

Eukaryotic cell lines

Policy information about cell lines

Cell line source(s)	Both primary glioblastoma cells used in the study were obtained from the brain tumor tissue from the patients at Copenhagen University Hospital. After, the cell lines were passaged subcutaneously in the flanks of NOG mice and isolated from xenografts. U87MG cell line was received from the manufacturer (ATCC).
Authentication	Cell lines lines were routinely authenticated using ATCC STR profiling.

Mycoplasma contamination	All cell lines were routinely tested for mycoplasma with tests showing absence of contamination.
Commonly misidentified lines (See ICLAC register)	Used cell lines do not belong to the misidentified cell lines.

Animals and other organisms

Policy information about studies involving animals; ARRIVE guidelines recommended for reporting animal research

Laboratory animals	Female NMRI-Nude mice (Taconic Biosciences, # NMRINU-F) aged 7-9 weeks were used for the present study.
Wild animals	Wild animals were not involved in the present study.
Field-collected samples	The present work does not involve field-collected samples.
Ethics oversight	All experimental procedures involving animals were conducted under the approval of institutional boards for ethical use of experimental animals (Copenhagen University and Technical University of Denmark) and the Danish Animal Experiments Inspectorate. Glioblastoma tissues, which were the source of cells used for xenografts, was obtained from patients in accordance with the guidelines outlined by the Danish Ethical Committee and the Danish Data Protection Agency (H-3-209-136_63114).

Note that full information on the approval of the study protocol must also be provided in the manuscript.
